# A Case of Alternating Oxaluria and Phosphaturia

**Published:** 1913-03

**Authors:** C. W. J. Brasher

**Affiliations:** Hon. Medical Registrar, Bristol Royal Infirmary.


### A CASE OF ALTERNATING OXALURIA AND PHOSPHATURIA.

BY

C. W. J. Brasher, M.D., Ch.B. Bristol,

*Hon. Medical Registrar, Bristol Royal Infirmary.*

---

In the preceding paper Professor Edgeworth has recorded an interesting case illustrating this very rare condition, and he has asked me to give details of a similar case also occurring in a male patient.

Mr. Charles M., aged 46, estate agent, a short, stout man who takes no exercise, has been under my care at intervals since 1902.

He is said to have had signs of early phthisis at the age of 20, and went to South Africa for two years. There is no history of gonorrhœa or syphilis. The patient is most erratic in his habits, often working and travelling day and night, taking food at irregular intervals, and either eating heavy meals and drinking stimulants freely and indiscriminately, sometimes to excess, or else subsisting on a diet of bread and milk and soda water.

For more than twenty years he has suffered from intermittent attacks of hæmaturia with much lumbar pain, chiefly on the right side, dysuria and frequently urethritis, but I have never found gonococci in the urethral discharge.

Before an attack of hæmaturia the urine is very acid, scanty, and highly coloured, and always deposits amorphous urates and a large amount of calcium oxalate crystals: during an attack the urine resembles that of a case of acute nephritis, but there are no casts, and the blood corpuscles are mixed with numerous very large "envelope" crystals.

These attacks of oxaluria invariably follow a period of excessive exertion with irregular and indigestible meals, culminating in an attack of acute dyspepsia, with vomiting, frontal headache, and a slight rise of temperature to 100° or 101°.

The heart sounds are normal, but he has considerable emphysema for a man of 46. The blood-pressure varies between 135 and 150 mm.Hg. These attacks of acute dyspepsia and oxaluria are immediately followed by the excretion of a much larger quantity of urine of low specific gravity containing a large amount of amorphous phosphates, so that the urine is, as the patient says, "as white as milk."

This phosphaturia invariably follows the other condition whether the patient takes alkaline medicines or not, but it should be said that he drinks Vichy water when the oxaluria occurs, and during the dyspeptic stage he takes nothing but milk foods and a large quantity of soda water. It seems probable, therefore, that the milk is largely responsible for the presence of an excess of calcium phosphate.

Several medical men have diagnosed renal calculus during these attacks of renal pain and hæmaturia, and on two occasions I have taken him to Mr. James Taylor, but the skiagrams have shown nothing abnormal.

One interesting point which I have observed on several occasions is that as the oxaluria is subsiding the large "envelope" crystals are gradually replaced by crystals of the "dumb-bell" variety; this may be due to some physical alteration in the urine, such as the presence of an increased quantity of mucus or an alteration of the calcium-magnesium ratio.

I have seen the patient quite recently, and he tells me that he has had no recurrence of hæmaturia and renal pain for more than a year, probably because he is more careful about his diet—he now avoids port and champagne, and still drinks a considerable quantity of Vichy water; he also informs me that he has not observed any deposit of phosphates.

It is generally admitted that oxalic acid in the urine is chiefly derived from various articles of food, notably cocoa, tea, spinach, rhubarb, and strawberries. In healthy individuals it is probable that food is the only source of oxalic acid.

Rendle Short[1] has proved that the ingestion of rather large quantities of fruit salad and rhubarb will produce a copious deposit of large oxalate crystals and urethral pain on micturition, and that in a case of oxalic acid poisoning the quantity excreted during 24 hours was ten times the normal amount.

Neuberg[2] says, " Klemperer and Tritschler have been able to prove conclusively in numerous cases a fact which had already been emphasised by Fürbringer, that a urine may fail to yield a sediment and yet be relatively rich in oxalic acid, and, conversely, a urine may yield an abnormal sediment and yet be relatively poor in the acid. For the separation of crystals among which may be crystals of calcium carbonate as well as of oxalate (Dunlop) depends less on the absolute amount of the acid than on certain physical and chemical features—the acidity of the urine, the ratio of the bases calcium and magnesium, with which the oxalic acid is combined, to each other, and on the presence of alkalis. That calcium oxalate, itself insoluble, might remain dissolved in urine was formerly referred to the acidity caused by acid sodium phosphate. In reality, according to Klemperer and Tritschler, yet another factor has to be considered, namely the ratio of the lime to the magnesia."

There seems little doubt, however, that in abnormal conditions of the digestive tract endogenous oxalic acid may be produced from carbohydrates, proteids, gelatin, and also from kreatin and kreatinin, as the result of digestive disturbances.

[1] Von Noorden, *Metabolism and Practical Medicine*, i. 148.

[2] *Ibid.*, iii. 1046.

This case suggests that the acute dyspepsia resulted in the production of a large amount of endogenous oxalic acid from the proteids and carbohydrates contained in the food, as well as the increased solution of exogenous oxalates in the highly acid gastric contents.

Unfortunately, I have never had an opportunity of examining the vomit, but the patient says it is intensely acid.

